# Study on the mechanism of *Xanthoceras sorbifolia* Bunge oil in the treatment of Alzheimer’s disease by an integrated “network pharmacology-metabolomics” strategy

**DOI:** 10.1080/07853890.2025.2499700

**Published:** 2025-05-08

**Authors:** Lijing Du, Yuanfang Sun, Yu Gan, Leqi Wang, Xinyi Li, Shikai Yan, Xue Xiao, Shasha Li, Huizi Jin

**Affiliations:** ^a^ Shanghai Key Laboratory for Molecular Engineering of Chiral Drugs, School of Pharmacy, Shanghai Jiao Tong University, Shanghai, China; ^b^ The Second Affiliated Hospital of Guangzhou University of Chinese Medicine, Guangzhou, China; ^c^ School of Clinical Medicine, Chengdu University of Traditional Chinese Medicine, Chengdu, China; ^d^ Institute of Traditional Chinese Medicine, Guangdong Pharmaceutical University, Guangzhou, China; ^e^ Jiyuan Neurohealth Industry Research Institute of Guangdong Pharmaceutical University, Jiyuan, China

**Keywords:** *Xanthoceras sorbifolia* Bunge oil, Alzheimer’s disease, network pharmacology, molecular docking, sphingolipid metabolism, inflammation

## Abstract

**Background:**

*Xanthoceras sorbifolia* Bunge oil (XSBO) has garnered significant interest from researchers due to its distinctive anti-Alzheimer’s disease (AD) properties. However, the underlying molecular mechanism remain unclear. This study aims to investigate the potential mechanisms by which XSBO may exert therapeutic effects on AD by employing a combination of network pharmacology analysis and experimental validation.

**Methods:**

The chemical composition and absorbed compounds of XSBO were identified using GC-MS and LC-MS. Network pharmacology analysis was performed using various computational tools to identify hub genes and construct compound-target-pathway networks. Subsequently, both *in vitro* and *in vivo* experiments were conducted to confirm the mechanisms by which XSBO may treat AD.

**Results:**

The results identified 43 active compounds in XSBO, targeting a total of 223 genes, of which 191 were associated with AD. Network analysis indicated that the active constituents in XSBO, such as 9,12-octadecadienoic acid, linoelaidic acid and 11-octadecenoic acid, interact with targets including MAPK1, MAPK3, AKT1, RXRA, RXRB, PPARD and PPARA to modulate inflammation-related signalling pathways and the sphingolipid signalling pathway. *In vitro* investigations corroborated that XSBO can significantly influence the viability of Aβ25-35-induced SH-SY5Y cells *via* the MAPK pathway.

**Conclusions:**

This study demonstrated that XSBO has the potential to mitigate inflammation network disorders through the MAPK pathway and to restore sphingolipid metabolite levels in AD rats, thereby laying a groundwork for future studies.

## 1. Introduction

Alzheimer’s disease (AD) is a chronic and progressive neurodegenerative disorder, characterized by irreversible cognitive impairments, changes in personality and behaviour, loss of functional abilities and other dementia-related symptoms. According to the World Alzheimer Report 2019, over 50 million individuals worldwide have been diagnosed with AD, and this number is projected to rise substantially to 150 million by 2050 [1]. Despite this, our understanding of the factors influencing the onset and progression of AD remains limited. The pathological changes observed in AD are complex, involving the aggregation of amyloid-beta (Aβ), hyperphosphorylation of tau protein, cholinergic system dysfunction, neuroinflammation and oxidative stress [2–5]. Currently, the primary therapeutic strategy for AD involves intervention of acetylcholinesterase inhibitors, such as galantamine and donepezil. These agents demonstrate limited effectiveness in alleviating cognitive and behavioural symptoms and do not have the capacity to prevent, halt, or reverse the progression of AD [6]. Given the intricate pathology of AD, the traditional therapeutic paradigm of ‘one drug-one target’ or ‘single-target drug’ is insufficient to achieve optimal therapeutic outcomes. Consequently, there is a growing interest among researchers in the exploration of natural products for drug development. The structural complexity and chemical diversity inherent in natural products position them as significant contributors to drug discovery and development.

*Xanthoceras sorbifolia* Bunge, a distinctive woody shrub belonging to the Sapindaceae family, is indigenous to China and is recognized for its substantial oil content [7]. This species is widely employed in the management of various medical conditions, including rheumatoid arthritis, cognitive impairments and paediatric enuresis, owing to its extensive therapeutic properties [8–10]. As an oil crop, the kernel of *Xanthoceras sorbifolia* Bunge is abundant in lipids, constituting 55–65% of its composition, with a significant proportion-predominant presence of polyunsaturated fatty acids, particularly linoleic and oleic acids [11]. *Xanthoceras sorbifolia* Bunge oil (XSBO), traditionally used as an edible oil in parts of China, has the medicinal potential of anti-oxidative and anti-inflammatory [12,13]. Recently, XSBO was found to be effective in enhancing the memory function in male mice [14]. The beneficial effects of XSBO are likely attributable to its high oleic acid content and the presence of the unique long-chain fatty acid, cis-15-tetracosenoic acid (nervonic acid). Nervonic acid is a critical component of biological membranes, playing a vital role in the development and maintenance of human brain nerve cells and tissues, and facilitating tissue repair and neuronal regeneration [15]. Consequently, XSBO has the potential to function as a complementary and alternative resource for enhancing learning and memory functions. Nonetheless, existing research on the behavioural or cognitive effects of XSBO in AD models is limited and remains at a nascent stage.

XSBO comprises complex components associated with various pathological targets and pathways, which presents challenges in comprehending its effects. Network pharmacology, which integrates cheminformatics, systems biology, traditional pharmacology and high-throughput data analysis, provides a robust and reliable approach for elucidating the intricate relationships among compounds, diseases and targets, thereby aiding the identification of underlying molecular mechanisms [16,17]. Additionally, molecular docking is an effective bioinformatics tool for validating interactions between candidate compounds and targets [18,19]. To date, over 70 approved drugs have been discovered by virtual screening, molecular docking and other computational techniques [20]. Thus, the aim of this study was to investigate the pharmacological mechanisms of XSBO in the context of AD using network pharmacology and experimental validation, thereby providing a theoretical framework for the prevention and management of AD.

## 2. Materials and methods

### 2.1. Chemicals and reagents

XSBO was provided by Shenzhen JustHerb Biopharmaceutical Co., Ltd. Methoxyamine hydrochloride (Lot#BCCD8751), HPLC-grade pyridine (99.9%) (Lot#SHBL0433) and N-methyl-Ntrimethylsilyltrifluoroacetamide (MSTFA) with 1% trimethylchlorosilane (TMCS) (Lot#BCCC9855) were obtained from Sigma Aldrich (St. Louis, MO, USA). Acetonitrile, methanol and Isopropanol were obtained from Merck (Merck, Germany). Analytical grade Methyl tert‑butyl ether (MTBE) was purchased from Sigma (St. Louis, MO).

### 2.2. Animal

A total of 44 male Sprague-Dawley (SD) rats (SPF, 260 ± 20 g) were procured from *Guangdong Vital River Laboratory Animal Technology Co., Ltd.* The animal experiments and procedures were reviewed and approved by the *Ethics Committee of Guangdong Provincial Hospital of Chinese Medicine* (ethics approval number: 2022016). All experimental procedures followed the ARRIVE guidelines (https://arriveguidelines.org/arrive-guidelines). Of these, 34 rats were randomly allocated into three groups, including control group (*n* = 10), model group (*n* = 12), XSBO treatment group (8.4 g/kg/d; *n* = 12). The daily oral dose of XSBO was determined based on the Dietary Guidelines for Chinese Residents [21], with 8.4 g/kg/day identified as the optimal dosage from our previous research. The drugs were administered orally *via* gavage once daily over a 4-week period. Starting from the 20th day, scopolamine (3 mg/kg) was injected into each group, except the control group, 30 min post-dosing. Rats in the control group received intraperitoneal injections of an equivalent volume of normal saline solution. An additional cohort of 10 rats was utilized to identify the absorbable compounds of XSBO. These rats were administered XSBO (8.4 g/kg/d; *n* = 5) and water (*n* = 5) *via* gastric gavage, twice daily, for three consecutive days. Two hours following the oral administration of XSBO on the fourth day, serum containing the drug was collected from the abdominal aorta.

### 2.3. GC-MS analysis of XSBO chemical compositions

A 10 μL aliquot of XSBO was dissolved in 400 μL of methanol and subsequently dried under a stream of nitrogen gas. For methoxyamination, 50 μL of methoxyamine hydrochloride in pyridine (20 mg/mL) was added to the sample, which was then subjected to sonication for 5 min. The sample was subsequently incubated in a water bath at 80 °C for 20 min. After cooling to room temperature, an equivalent volume of MSTFA containing 1% TMCS (50 μL) was added, and the mixture was maintained at 80 °C for 20 min. Following the cooling process, the sample was centrifuged at 13,000 rpm for 15 min at 4 °C.

The fatty acid methyl esters derived from XSBO were detected by an Agilent 7890 A-5975C gas chromatography-mass spectrometry (GC-MS) system (Agilent Technologies, Santa Clara, CA). The separation was achieved utilizing an Agilent HP-5MS 5% Phenyl Methyl Siloxane capillary column (30 *m* × 250 μm × 0.25 μm). The experimental conditions were as follows: the initial column temperature was maintained at 60 °C for 5 min, followed by an increase to 200 °C at a rate of 10 °C per minute, where it was held for 3 min. The temperature was then elevated to 240 °C at a rate of 4 °C per minute, and subsequently increased to 280 °C at a rate of 10 °C per minute, where it was maintained for 2 min. The sample was introduced in split mode with a split ratio of 10:1 and an injection volume of 1 μL.

The mass spectrometry parameters were set as follows: the ion source operated in electron ionization (EI) mode with an electron energy of 70 eV, the ion source temperature was set to 230 °C, the MS quadrupole temperature maintained at 150 °C, the interface temperature set at 260 °C, the front inlet purge flow rate was 3 mL/min, a full scan was conducted within a mass range of 30–550 amu, and a solvent delay of 7.0 min was implemented. Compounds were identified utilizing the NIST 14.0 mass spectra libraries, and the relative percentage content of each component was determined through the peak area normalization method.

### 2.4. Network pharmacology analysis

#### 2.4.1. Active compounds and target screening

The chemical composition of XSBO was analyzed by GC-MS. Subsequently, the SMILES structures of the compounds, retrieved from PubChem, were inputted into Swiss Target Prediction (http://www.swisstargetprediction.ch/) to forecast the potential targets of XSBO. The parameters were set to ‘Homo sapiens’ with a probability threshold exceeding 0.1. Data concerning targets associated with AD were sourced from the DisGeNET (https://www.disgenet.org/) and GeneCard (https://www.genecards.org/) databases, employing ‘Alzheimer’s disease’ as the search criterion. All target names were standardized *via* the UniProt database (https://www.uniprot.org/). The target genes common to both XSBO and AD were identified using the Venny2.1.0 (https://bioinfogp.cnb.csic.es/tools/venny/). To visually depict the interaction between the candidate compounds and potential targets, a compound-target network was constructed using Cytoscape 3.7.2.

#### 2.4.2. Construction of the PPI network

To evaluate the interaction relationships between target proteins, a protein-protein interaction (PPI) network was constructed by the STRING database (version 11.5; https://cn.string-db.org/), and visualized with Cytoscape 3.7.2. The research focused on the species ‘Homo sapiens’, and only targets with a high confidence score of 0.7 or above were considered. The Molecular Complex Detection (MCODE) algorithm (http://baderlab.org/Software/MCODE) was employed to identify virtual modules and key genes that form the stable structure of the network. The top 10 hub targets were selected by the maximal clique centrality (MCC) method within the CytoHubba plugin (http://apps.cytoscape.org/apps/cytohubba) [22].

#### 2.4.3. Gene ontology (GO) and Kyoto encyclopedia of genes and genomes (KEGG) analysis

GO enrichment analysis and KEGG pathway enrichment analysis were performed using the Metascape platform (version 3.5; https://metascape.org/gp/index.html#/main/step1), with ‘Homo sapiens’ as the species of interest. GO enrichment analysis encompassed biological processes (BP), cellular components (CC) and molecular functions (MF). Target genes from the top 10 KEGG signalling pathways, along with their corresponding components, were chosen to construct a component-target-pathway network, using Cytoscape 3.7.2.

### 2.5. GC-MS and LC-MS analysis of XSBO absorbable compounds

To acquire more comprehensive data on XSBO absorbable compounds, a combination of GC-MS and LC-MS techniques were used to analyze absorbable compounds in the serum. The protocol for sample preparation and instrumental parameters is detailed as follows.

**GC-MS Analysis**: A volume of 100 μL serum was dissolved in 400 μL of methanol and subsequently evaporated to dryness using a stream of nitrogen gas. The derivatization procedure and detection parameters for GC-MS were consistent with those outlined in Section 2.3.

**LC-MS Analysis:** A volume of 100 μL of serum containing the XSBO drug, as well as control serum, was dissolved in 300 μL of methanol. Following vortexing for 30 s, the mixture was centrifuged at 13,000 rpm and 4 °C for 15 min, followed by microfiltration. The samples were analyzed using a Waters ACQUITY UPLC system (Milford, USA) coupled with a Triple TOF 5600 mass spectrometer (AB Sciex, USA). Chromatographic separations were conducted using an Acquity UPLC BEH C18 column (100 mm × 2.1 mm; 1.7 μm) maintained at a temperature of 35 °C. The mobile phases comprised of 0.1% formic acid (A) and methanol (B), with a flow rate of 0.3 mL/min and a sample injection volume of 5 μL. The gradient elution program for the mobile phase was as follows: 0–2 min, 2–2% B; 2–3 min, 2–20% B; 3–9 min, 20–40% B; 9–18 min, 40–40% B; 18–20 min, 40–72% B; 20–25 min, 72–75% B; 25–40 min, 75–85% B; 40–42 min, 85–94% B; 42–50 min, 94–95%B; 50–52 min, 95–98%B; 52–56 min, 98–98%B.

Mass spectrometric detection was conducted using an electrospray ionization (ESI) source operating in both positive and negative ionization modes. The optimal parameters for the experiment included source gases 1 and 2, each maintained at 50 psi, a curtain gas at 50 psi, an interface heater temperature of 500 °C, a de-clustering voltage of 80 V and ion spray voltages of 5500 V and −4500 V for positive and negative ion modes, respectively. Data acquisition for each sample was conducted over a mass range of 50 to 1500 Da.

The absorbed chemical compounds of XSBO were identified through comparative analysis of mass spectrometry data obtained from control rat serum, XSBO-administered rat serum and XSBO extracts. Peaks were detected in both the XSBO-containing rat serum and XSBO extract samples at identical retention times, while they were absent in the control rat serum or the peak intensities in the XSBO-containing rat serum were higher twice or more than those in the control serum, suggesting the presence of absorption compounds.

### 2.6. Molecular docking

The optimal 3D conformation of the primary targets was obtained from the Protein Data Bank (PDB) repository (http://www.rcsb.org/) and stored in PDB format. The 3D structural file of the potential compound was retrieved from the PubChem database (http://pubchem.ncbi.nlm.nih.gov). Ligands and receptors were prepared using AutoDock Tools software (version 1.5.6). Molecular docking was performed by Autodock Vina 1.2.2 (http://autodock.scripps.edu/). The binding energy value serves as an indicator of the binding strength and activity between ligands and receptors, with lower values indicating a stronger binding capacity. Generally, when the affinity energy score (kcal/mol) is less than −5 in the docking interaction between the ligand molecule and the target receptor, the binding activity is deemed favourable.

### 2.7. Lipidomic analysis

In alignment with previous research findings, with slight modifications [23], 10 mg of brain tissue was accurately weighed and homogenized with 400 μL of 75% methanol. Subsequently, 1 mL of MTBE was addedintroduced and the mixture was centrifuged at 13,000 rpm for 15 min at 4 °C. To facilitate the separation of the upper and lower layers, 300 μL of water was added to the tissue homogenates. After vortexing for 30 s and incubating for 10 min, the samples were centrifuged again at 13,000 rpm for 15 min at 4 °C. A 300 μL aliquot from the upper layer was then extracted for lipidomics analysis.

Lipidomic profiling was conducted utilizing a Waters ACQUITY UPLC system (Milford, USA) coupled with a Triple TOF 5600 mass spectrometer (AB Sciex, USA). Chromatographic separations were carried out on an Acquity UPLC CSH C18 column (100 mm × 2.1 mm; 1.7 μm) maintained at a temperature of 35 °C. The mobile phases employed in this study consisted of two solutions: Solution A, which was a mixture of 60% acetonitrile and 40% water, supplemented with 10 mM ammonium formate and 0.1% formic acid; Solution B, which was composed a mixture of 90% isopropanol and 10% acetonitrile, also containing 10 mM ammonium formate and 0.1% formic acid. The flow rate was set at 0.4 mL/min, with a sample injection volume of 5 μL. A gradient elution program of mobile phase was as follows: 0–2 min, 0–10% B; 2–4 min, 10–40% B; 4–6 min, 40–45% B; 6–10 min, 45–48% B; 10–25 min, 48–52% B; 25–30 min, 52–58% B; 30–35 min, 58–80% B; 35–38 min, 80–100% B.

MS detection was performed using an electrospray ionization (ESI) source, operating in both positive and negative ionization modes. The optimized parameters were as follows: source gas 1 at 50 psi, source gas 2 at 50 psi, curtain gas at 50 psi, an interface heater temperature of 500 °C, a de-clustering voltage of 80 V and ion spray voltages of 5500 V and −4500 V for positive and negative modes, respectively. Data acquisition for each sample was conducted over a mass range of 50 to 1500 Da. Lipid identification was facilitated using MS-DIAL 4.70 software in conjunction with the LipidBlast database to match the MS/MS spectra of each feature. The preprocessed dataset was subsequently subjected to analysis using Simca-P 14.0 Software for multivariate statistical evaluation. Orthogonal partial least-squares discriminant analysis (OPLS-DA) was utilized to distinguish between the various experimental groups. The OPLS-DA models were validated by permutation test (200 times). Lipids were considered differentially expressed if they exhibited a Variable Importance in Projection (VIP) score exceeding 1 and a *p*-value below 0.05.

### 2.8. Biochemical indicators detection

The prefrontal cortices of each rat were collected and homogenized in phosphate-buffered saline (PBS) at a weight-to-volume ratio of 1:9. The supernatants were isolated following centrifugation. In accordance with the manufacturer’s protocol, concentrations of interleukin-1β (IL-1β), tumour necrosis factor α (TNF-α) and nuclear factor kappa B (NF-κB) were quantified using ELISA kits provided by Shanghai Enzyme-linked Biotechnology Co., Ltd.

### 2.9. Targeted inflammatory mediator analysis

Prefrontal cortices from rats were collected, homogenized in PBS at a weight-to-volume ratio of 1:10, and centrifuged at 13,000 rpm for 15 min at 4 °C to obtain the supernatant. Subsequently, 200 μL of the supernatant was mixed with 400 μL of water containing 4% v/v phosphoric acid using a vortex mixer for 2 min, followed by centrifugation at 13,000 rpm for 10 min at 4 °C. The purification and enrichment of analytical samples were performed using solid-phase extraction with Waters Oasis HLB 1 cc (30 mg) Cartridges (Waters, Milford, MA) followed by nitrogen drying. Subsequently, the samples were reconstituted in 200 μL of acetonitrile, and filtered through 0.22-μm membrane filters. The precision (assessed by injecting a quality control sample six times), repeatability (evaluated by continuous injection of six parallel samples), and stability (analyzed at 0, 2, 4, 8, 12 and 24 h) of the analytical method were examined during method validation. Additionally, an appropriate volume of methanol was added to 84 inflammatory mediator standards to prepare a series of mixed standard solutions with concentrations ranging from 0.39 to 400 μg/mL. Compounds information are listed in Table S1.

Ultrahigh-performance liquid chromatography coupled with a triple quadrupole mass spectrometer (Xevo-TQ-XS, Waters, USA) was employed for the analysis of inflammatory mediator. Separation was performed on a Waters Acquity UPLC BEH C18 column (100 mm × 2.1 mm, 1.7 µm) maintained at a temperature of 35 °C. The mobile phase comprised of water with 0.1% formic acid (mobile phase A) and acetonitrile with 0.1% formic acid (mobile phase B). The gradient elution program was implemented as follows: 0–4 min, 25–35% B; 4–20 min, 35–85% B; 20–23 min, 85–95% B; 23–25 min, 95–95% B. An injection volume of 5 μL was utilized, with a flow rate maintained at 0.3 mL/min. The mass spectrometry parameters were set as follows: electrospray ionization source, multiple reaction monitoring (MRM) in negative ion mode, capillary voltage set to 2.5 kV, desolvation temperature at 500 °C, desolvation gas flow rate of 800 L/h, cone gas flow rate of 150 L/h, and collision gas flow rate of 0.15 mL/min.

### 2.10. Cell culture and treatments

SH-SY5Y human neuroblastoma cells were maintained in DMEM/F12 medium supplemented with 10% foetal bovine serum and 100 U/ml penicillin/streptomycin. To establish an AD cell model, the cells were pre-treated with varying concentrations of Aβ25-35 (10, 20, 40, 80 μM) for 24 and 48 h, respectively. Cell viability was assessed using the xCELLigence RTCA System, CCK8 assay, flow cytometry with Annexin V-FITC/propidium iodide (PI) staining, and Hoechst 33258 staining. Cells exhibiting bright blue fluorescence upon Hoechst staining were identified as apoptotic, characterized by condensed chromatin or fragmented nuclei. Following the determination of optimal concentration and incubation period, cells were pre-treated with XSBO drug-containing serum (2.5%, 5%, or 10%) for 2 h prior to exposure to Aβ25-35 for 24 h. Cellular lysates were subsequently collected for ELISA to measure Aβ42, phosphorylated Tau, TNF-α and IL-1β as well as for western blot analysis.

### 2.11. Western blotting

The RIPA lysis buffer was employed to extract total protein from cellular samples. The extracted proteins were subsequently separated using a 10% SDS-PAGE gel and transferred onto PVDF membranes. The membranes were then blocked for 1 h using a 5% skimmed milk solution. Following this, the membranes were incubated overnight at 4 °C with primary antibodies specific to ERK (dilution 1:1000, Cell Signaling Technology), p-ERK (dilution 1:2000, Cell Signaling Technology), P38 (dilution 1:1000, Cell Signaling Technology), p-P38 (dilution 1:2000, Cell Signaling Technology) and Tubulin (dilution 1:1000, Cell Signaling Technology). Subsequently, the membranes were incubated for 1 h at room temperature with HRP-conjugated anti-rabbit IgG secondary antibodies (dilution 1:4000, Cell Signaling Technology). Protein detection and analysis were conducted using the Bio-Rad ChemiDoc Touch Imaging System.

### 2.12. Statistical analysis

Statistical analysis of the various groups was performed using SPSS Statistics 18.0 (SPSS, USA), with data presented as mean ± standard deviation. The students t-test was employed for comparing means between groups under the assumption of normal distribution, whereas the Mann-Whitney test was applied for non-normally distributed data. A *p*-value of less than 0.05 was considered indicative of statistically significant differences.

## 3. Results

### 3.1. Chemical compositions of XSBO

**Figure 1.**
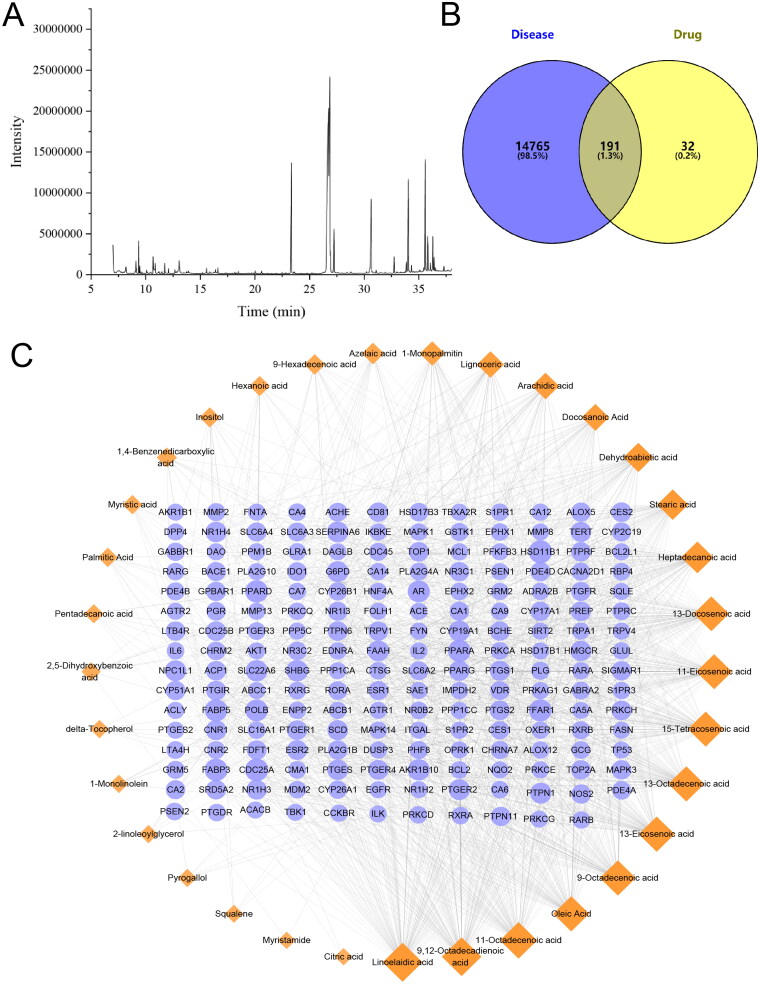
(a) GC-MS chromatogram of XSBO; (b) a Veen diagram of common gene targets of AD and XSBO; (c) the network diagram of active ingredient-common target. Diamond-shaped nodes represent components, circular-shaped nodes represent targets, and the edges represent the interactions between components and targets. The colour and size depth of the nodes correspond to the degree value.

The chemical composition of XSBO was analyzed using GC-MS, with the resulting chromatogram depicted in Figure 1a and the detailed compositional data presented in Table 1. The findings reveal that XSBO is abundant in unsaturated fatty acids, such as 9,12-octadecadienoic acid, 9-octadecenoic acid, palmitic acid, 13-docosenoic acid, 1-monolinolein, 15-tetracosenoic acid and oleic acid. These experimental results are consistent with previously published studies [24].

**Table 1.** Analysis of XSBO composition and absorbed compounds.

No.	Retention time (min)	Compound	Molecular formula	CAS	Relative percentage content (%)	GC-MS	LC-MS
1	7.88	2,4,6-octatrienoic acid	C_8_H_10_O_2_	5205-32-3	0.15		
2	9.44	lactic acid	C_3_H_6_O_3_	50-21-5	0.47		
3	9.55	hexanoic acid	C_6_H_12_O_2_	142-62-1	0.07		
4	9.66	glycolic acid	C_2_H_4_O_3_	79-14-1	0.07		
5	15.21	glycine	C_2_H_5_NO_2_	56-40-6	0.04		
6	15.97	2-hexenedioic acid	C_6_H_8_O_4_	2583-24-6	0.13		
7	16.38	5-oxoproline	C_5_H_7_NO_3_	98-79-3	0.36		
8	17.36	pyrogallol	C_6_H_6_O_3_	87-66-1	0.08		
9	19.45	azelaic acid	C_9_H_16_O_4_	123-99-9	0.12		
10	19.75	methylmaleic acid	C_5_H_6_O_4_	498-23-7	0.05		
11	19.92	citric acid	C_6_H_8_O_7_	77-92-9	0.06		
12	20.04	myristic acid	C_14_H_28_O_2_	544-63-8	0.23	√	
13	20.62	inositol	C_6_H_12_O_6_	87-89-8	0.10	√	
14	21.48	pentadecanoic acid	C_15_H_30_O_2_	1002-84-2	0.05		
15	22.78	9-hexadecenoic acid	C_16_H_30_O_2_	10030-73-6	0.08		
16	23.34	palmitic acid	C_16_H_32_O_2_	57-10-3	9.37	√	
17	23.54	7-methyl-3,4-octadiene	C_9_H_16_	37050-05-8	0.12		
18	23.54	(Z,Z)-3,6-nonadienal	C_9_H_14_O	21944-83-2	0.12		
19	25.22	heptadecanoic acid	C_17_H_34_O_2_	506-12-7	0.09		
20	26.72	9,12-octadecadienoic acid	C_18_H_32_O_2_	2197-37-7	31.88	√	
21	26.84	9-octadecenoic acid	C_18_H_34_O_2_	112-79-8	23.32	√	
22	27.23	stearic acid	C_18_H_36_O_2_	57-11-4	3.64	√	
23	27.62	oleic acid	C_18_H_34_O_2_	112-80-1	0.16	√	
24	28.00	xanthurenic acid	C_10_H_7_NO_4_	59-00-7	0.09		
25	28.42	linoelaidic acid	C_18_H_32_O_2_	506-21-8	0.12		√
26	29.65	13-octadecenoic acid	C_18_H_34_O_2_	693-71-0	0.09		
27	30.10	dehydroabietic acid	C_20_H_28_O_2_	1740-19-8	0.04		
28	30.63	13-eicosenoic acid	C_20_H_38_O_2_	17735-94-3	6.56	√	
29	30.74	11-eicosenoic acid	C_20_H_38_O_2_	5561-99-9	0.20	√	√
30	31.10	arachidic acid	C_20_H_40_O_2_	506-30-9	0.24		
31	31.45	myristamide	C_14_H_29_NO	638-58-4	0.06		
32	33.33	2,5-dihydroxybenzoic acid	C_7_H_6_O_4_	490-79-9	0.11		
33	33.86	1-monopalmitin	C_19_H_38_O_4_	542-44-9	1.26		
34	34.04	13-docosenoic acid	C_22_H_42_O_2_	1072-39-5	6.37		√
35	34.33	docosanoic Acid	C_22_H_44_O_2_	112-85-6	0.46		
36	34.72	11-octadecenoic acid	C_18_H_34_O_2_	693-72-1	0.10		√
37	35.46	2-linoleoylglycerol	C_21_H_38_O_4_	3443-82-1	0.39		
38	35.59	1,4-benzenedicarboxylic acid	C_8_H_6_O_4_	100-21-0	6.70		
39	35.81	1-monolinolein	C_21_H_38_O_4_	26545-74-4	2.75		
40	36.28	15-tetracosenoic acid	C_24_H_46_O_2_	506-37-6	2.16		√
41	36.40	squalene	C_30_H_50_	111-02-4	0.84		
42	36.51	lignoceric acid	C_24_H_48_O_2_	557-59-5	0.31		
43	37.31	delta-tocopherol	C_27_H_46_O_2_	119-13-1	0.39		

### 3.2. Network pharmacology analysis of XSBO treating AD

A total of 223 potential targets associated with XSBO were predicted by the Swiss Target Prediction database. By integrating AD-related disease targets from the DisGeNET and GeneCard databases, 14956 AD-related targets were identified. To investigate the potential relationship between XSBO and AD, the Venny 2.1 online tool was employed to identify common targets, resulting in the identification of 191 common targets, as illustrated in Figure 1b. The active components and common targets were subsequently imported to construct a compound-disease target interaction network (Figure 1c). Through network analysis, nodes with degree values exceeding 70 were identified as the core compounds of XSBO in the treatment of AD. The results indicated that several fatty acids, such as linoelaidic acid, 9,12-octadecadienoic acid, 11-octadecenoic acid, oleic Acid, 9-octadecenoic acid, 13-octadecenoic acid, 13-eicosenoic acid, 15-tetracosenoic acid, 11-eicosenoic acid, 13-docosenoic acid, may play a significant role in the resistance mechanisms associated with AD.

**Figure 2.**
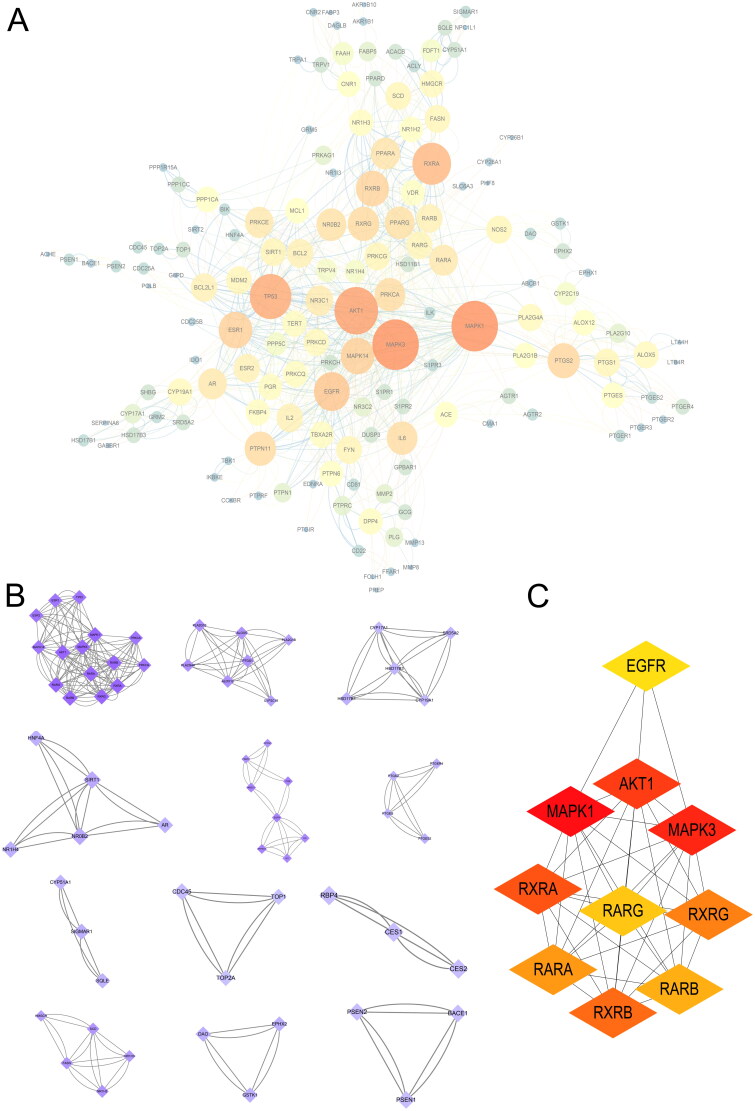
(a) PPI Network of target proteins of XSBO by cytoscape; (b) key modules based on the PPI network by the MCODE plugin; (c) top 10 hub gene identified by cytohubba. The different colours and sizes of the PPI network nodes represented the degree of interactions between the targets. The higher the degree, the bigger the nodes and the darker the node color.

Furthermore, 191 common targets were imported into the STRING database to establish a PPI network, with a confidence score exceeding 0.7 (Figure 2a). To achieve a more comprehensive understanding of the PPI network, gene cluster analysis and core gene scanning were carried out utilizing the MCODE plug-in. This analysis identified a total of 12 significant gene clusters, the results of which are presented in Figure 2b and Table 2. The top 10 targets within the network were identified, as illustrated in Figure 2c. The PPI network and MCODE plugin were employed to analyze the critical modules derived from the hub genes, thereby elucidating the action mechanisms of the core targets (MAPK1, MAPK3, AKT1, RXRA, RXRB, RARA, RARB, RARG, EGFR) of XSBO.

**Table 2.** List of MCODE modules.

No.	MCODE score	Gene	Nodes	Edges
1	10	ESR2, MAPK14, RARA, RXRG, MAPK3, TP53, RXRB, RARG, AKT1, ESR1, RXRA, MAPK1, RARB, PRKCA, PRKCG	15	140
2	5.333	PLA2G1B, PLA2G4A, ALOX5, PTGS1, CYP2C19, ALOX12, PLA2G10	7	32
3	4.5	CYP17A1, CYP19A1, HSD17B3, SRD5A2, HSD17B1	5	18
4	4	FASN, NR1H2, NR1H3, SCD, HMGCR	5	16
5	3.5	SIRT1, HNF4A, NR0B2, AR, NR1H4	5	14
6	3.429	IL2, FKBP4, NR3C1, IL6, PPP5C, EGFR, PTPN11, PGR	8	24
7	3.333	PTGER4, PTGES, PTGS2, PTGES2	4	10
8	3	GSTK1, DAO, EPHX2	3	6
9	3	CYP51A1, SIGMAR1, SQLE	3	6
10	3	TOP2A, CDC45, TOP1	3	6
11	3	CES2, RBP4, CES1	3	6
12	3	PSEN1, BACE1, PSEN2	3	6

**Figure 3.**
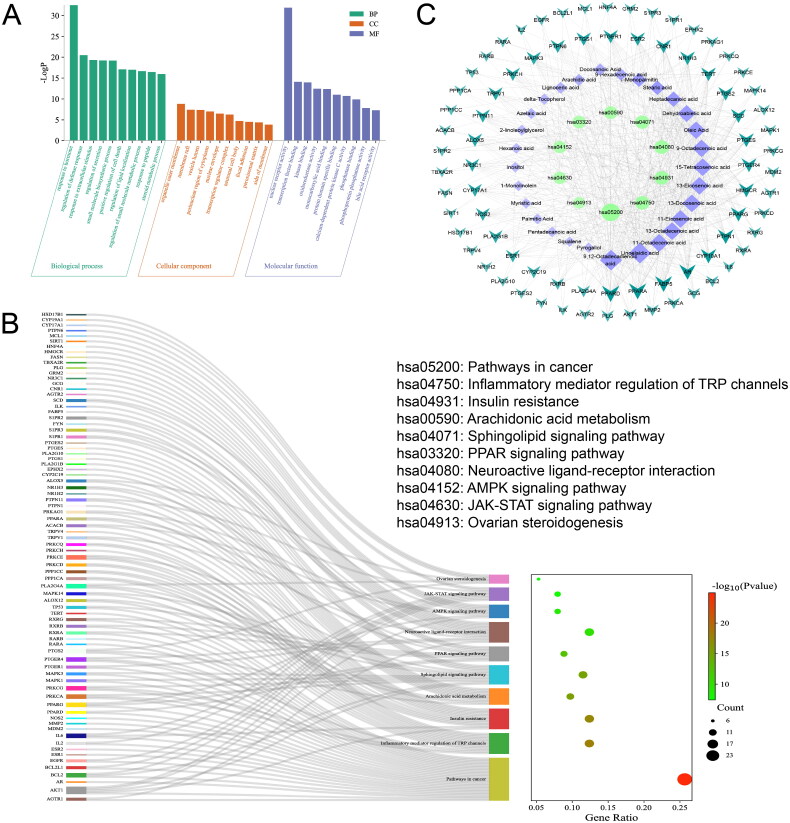
(a) GO Enrichment column diagram; (b) bubble chart of KEGG pathway analysis; (c) the ‘compound-target-pathway’ network of XSBO against AD.

The GO and KEGG enrichment analysis were performed using the target genes within the PPI network. The top 10 GO annotations (BP, CC and MF) were chosen based on a significance threshold of *p* < 0.05. As shown in Figure 3a, the BP category included processes such as response to hormone, cellular response to hormone stimulus, and cellular response to lipid, among others. The CC category encompassed elements such as organelle outer membrane, outer membrane and Bcl-2 family protein complex, among others. Concurrently, the MF category demonstrated diverse activities, including nuclear receptor activity, ligand-activated transcription factor activity and nuclear steroid receptor activity. The KEGG pathway enrichment analysis identified 167 signalling pathways, with the top 10 pathways depicted in Figure 3b. These pathways mainly involved pathways in cancer, inflammatory mediator regulation of TRP channels, insulin resistance, arachidonic acid metabolism, sphingolipid signalling pathway, PPAR signalling pathway, neuroactive ligand-receptor interaction, AMPK signalling pathway, JAK-STAT signalling pathway and ovarian steroidogenesis.

To elucidate the regulation of core targets and signalling pathways pertinent to AD treatment, we employed Cytoscape 3.7.2 to construct a compound-target-pathway interaction network. This network comprised of 118 nodes, including 29 compounds, 79 targets and 10 pathways, interconnected by 606 edges (Figure 3c). In this network, green circular nodes represent the top 10 significant signalling pathways, purple diamond nodes represent potential active components, and dark green V-shaped nodes represent key targets. The edges illustrate the interactions among these nodes, with the colour intensity and size of the nodes reflecting their degree values. Based on the network analysis, we hypothesize that compounds such as 9,12-octadecadienoic acid, linoelaidic acid, 11-octadecenoic acid, 13-octadecenoic acid and 11-eicosenoic acid exert regulatory role on inflammation-related signalling pathways and the sphingolipid signalling pathway. These effects are mediated through core target genes, including PPARD, PPARA, FABP5, AR and CYP19A1, among others.

### 3.3. The absorbed constituents from orally administered XSBO

**Figure 4.**
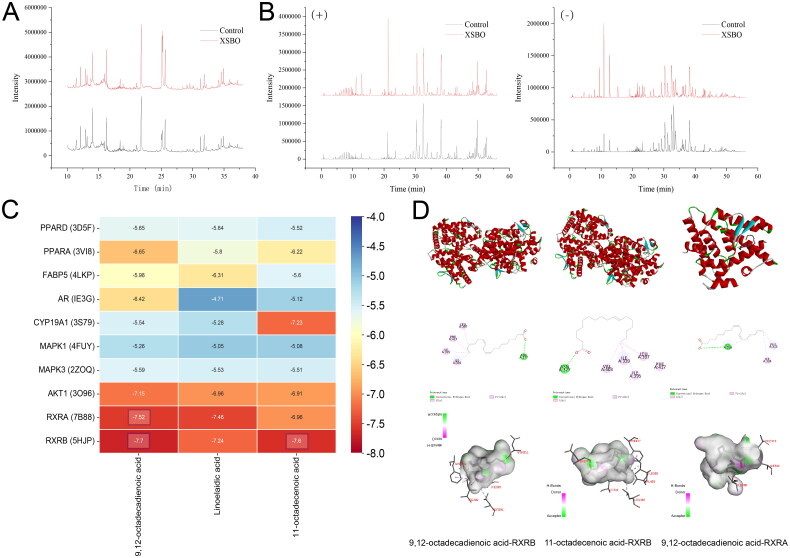
(a) GC-MS Chromatograms of absorbed constituents from orally administered XSBO; (b) LC-MS chromatograms of absorbed constituents from orally administered XSBO; (c) Molecular docking energy heat map; (d) The 2D and 3D molecular interaction diagrams of the best docking poses of compounds.

According to the theory of serum pharmacochemistry, the absorption of bioactive components into the bloodstream is considered as a prerequisite for the therapeutic efficacy of drug treatments [25]. To identify the key active constituents of XSBO as determined by network pharmacology analysis, the absorbed compounds were examined using LC-MS and GC-MS techniques (Figure 4a-b). Results indicated a total of 13 absorbed compounds were identified and summarized in Table 1, including inositol, palmitic acid, 9,12-octadecadienoic acid, 9-octadecenoic acid, stearic acid, oleic acid, 13-eicosenoic acid, 11-eicosenoic acid, myristamide,11-octadecenoic acid, 13-docosenoic acid, linoelaidic acid and 15-tetracosenoic acid. Notably, the majority of these compounds were also identified as potential active ingredients through network pharmacology predictions.

### 3.4. Molecular docking of active ingredients with key targets

To further investigate the binding affinity of the active constituents in XSBO to the core targets, molecular docking was performed. The top 3 active constituents, ranked according to the degree value within the compound-target-pathway network and identified as absorbed compounds of XSBO, were selected as ligands. Additionally, the top 5 target genes from the compound-target-pathway network and the top 5 hub genes identified *via* CytoHubba were selected as docking proteins. Figure 4c showed that the binding energies of 9,12-octadecadienoic acid, linoelaidic acid and 11-octadecenoic acid to most of the key target proteins were below − 5.0 kcal/mol, indicating, a strong binding interaction between the selected active constituents and the core proteins. The three compounds exhibiting the lowest binding energies (9,12-octadecadienoic acid-RXRB, 11-octadecenoic acid-RXRB, 9,12-octadecadienoic acid-RXRA) were selected for visualization, and are shown in Figure 4d.

### 3.5. XSBO can alleviate the disorders of sphingolipid metabolism caused by AD

**Figure 5.**
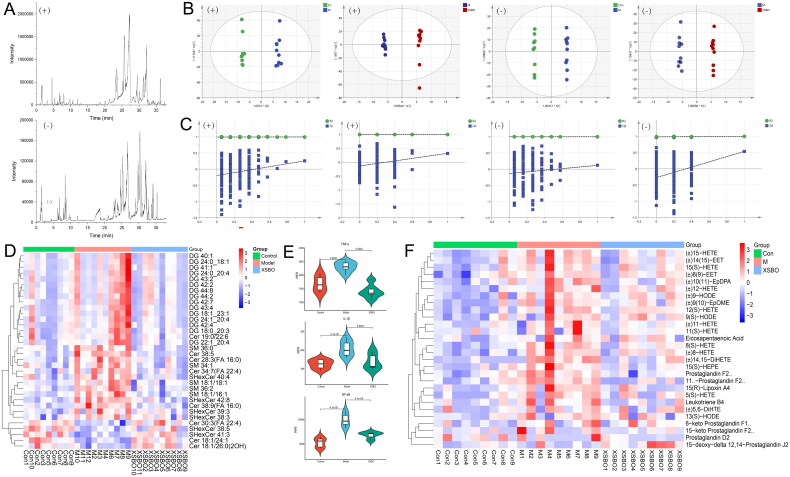
(a) Base peak chromatogram of lipidomics in positive and negative modes; (b) OPLS-DA score plots; (c) Results of permutation test (*n* = 200); (d) Clustering heat map analysis of differential lipids; (e) Effect of XSBO on inflammatory factors in rat brain tissue; (f) Changes of inflammatory mediators in the cerebral cortex of rats.

XSBO is abundant in polyunsaturated fatty acids, which are particularly prone to lipid peroxidation and oxidative stress, thereby influencing lipid degradation and composition. Additionally, 15-tetracosenoic acid, one of the most prevalent sphingolipid components, plays a crucial role as a structural molecule in the cell membrane and in the regulation of cellular events [26]. In the pathway enrichment analysis (Figure 3b), the sphingolipid signalling pathway emerged as one of the top canonical pathways. Alterations in sphingolipid content may contribute to the pathogenesis of AD. In the brains of AD patients, there is an observed increase in the content and expression of ceramides, sphingomyelins, glycosphingolipids, and their associated metabolic enzymes, such as sphingomyelinases [27]. To elucidate the impact of XSBO on the lipid profile of AD rats, we performed a comprehensive lipidomic analysis. The base peak chromatogram (BPC) of the lipidomic analysis is presented in Figure 5a. Lipids that satisfied both the OPLS-DA screening criteria (VIP > 1, Figure 5b) and *p* < 0.05 were identified as significantly different lipids (Table 3). The OPLS-DA models demonstrated robust and verifiable parameters, and a permutation test with 200 iterations confirmed that the models were not overfitted (Figure 5c). The heat plot for these differential lipids is shown in Figure 5d. Following the protocols described above, we noted that 5 potential sphingomyelins (SMs) biomarkers across the three groups. The levels of five SMs (SM 18:1/16:1, SM 18:1/18:1, SM 34:1, SM 36:0, SM 36:2) were significantly elevated in the model group compared to the control group, whereas XSBO supplementation resulted in a reduction of these lipids. These findings indicate that SM may serve as a pivotal target for AD therapy.

**Table 3.** The differential lipids regulated by XSBO.

ID	Compound name	Adduct	Retention time (min)	Formula	Class
1	Cer 38:5	[M-H]^-^	9.31	C_38_H_67_NO_4_	Cer
2	Cer 19:0/22:6	[M + H]^+^	10.48	C_41_H_71_NO_3_	Cer
3	Cer 28:3(FA 16:0)	[M + CH_3_COO]^-^	12.34	C_44_H_81_NO_5_	Cer
4	Cer 34:7(FA 22:4)	[M-H]^-^	12.61	C_56_H_89_NO_5_	Cer
5	Cer 38:9(FA 16:0)	[M + CH_3_COO]^-^	12.76	C_54_H_89_NO_5_	Cer
6	Cer 30:3(FA 22:4)	[M + CH_3_COO]^-^	12.80	C_52_H_89_NO_5_	Cer
7	Cer 18:1/24:1	[M-H]^-^	13.04	C_42_H_81_NO_3_	Cer
8	Cer 18:1/26:0(2OH)	[M-H]^-^	13.26	C_44_H_87_NO_4_	Cer
9	DG 42:7	[M + Na]^+^	11.88	C_45_H_74_O_5_	DG
10	DG 44:8	[M + Na]^+^	12.31	C_47_H_76_O_5_	DG
11	DG 18:0_20:3	[M + NH_4_]^+^	12.78	C_41_H_74_O_5_	DG
12	DG 42:4	[M + Na]^+^	13.06	C_45_H_80_O_5_	DG
13	DG 22:1_20:4	[M + NH_4_]^+^	13.12	C_45_H_78_O_5_	DG
14	DG 43:4	[M + Na]^+^	13.24	C_46_H_82_O_5_	DG
15	DG 24:1_20:4	[M + NH_4_]^+^	13.26	C_47_H_82_O_5_	DG
16	DG 40:1	[M + Na]^+^	13.38	C_43_H_82_O_5_	DG
17	DG 18:1_23:1	[M + NH_4_]^+^	13.43	C_44_H_82_O_5_	DG
18	DG 24:0_20:4	[M + NH_4_]^+^	13.43	C_47_H_84_O_5_	DG
19	DG 42:2	[M + Na]^+^	13.47	C_45_H_84_O_5_	DG
20	DG 41:1	[M + Na]^+^	13.55	C_44_H_84_O_5_	DG
21	DG 43:2	[M + Na]^+^	13.55	C_46_H_86_O_5_	DG
22	DG 24:0_18:1	[M + NH_4_]^+^	13.63	C_45_H_86_O_5_	DG
23	DG 44:2	[M + Na]^+^	13.68	C_47_H_88_O_5_	DG
24	SHexCer 42:8	[M-H]^-^	7.84	C_48_H_79_NO_12_S	SHexCer
25	SHexCer 38:5	[M-H]^-^	7.91	C_44_H_77_NO_12_S	SHexCer
26	SHexCer 40:4	[M-H]^-^	8.33	C_46_H_83_NO_12_S	SHexCer
27	SHexCer 38:3	[M-H]^-^	8.44	C_44_H_81_NO_11_S	SHexCer
28	SHexCer 39:3	[M-H]^-^	8.59	C_45_H_83_NO_11_S	SHexCer
29	SHexCer 40:3	[M-H]^-^	8.66	C_46_H_85_NO_11_S	SHexCer
30	SHexCer 41:3	[M-H]^-^	8.68	C_47_H_87_NO_12_S	SHexCer
31	SM 18:1/16:1	[M + H]^+^	8.50	C_39_H_77_N_2_O_6_P	SM
32	SM 34:1	[M + H]^+^	9.01	C_39_H_79_N_2_O_6_P	SM
33	SM 18:1/18:1	[M + H]^+^	9.05	C_41_H_81_N_2_O_6_P	SM
34	SM 36:2	[M + H]^+^	9.17	C_41_H_81_N_2_O_6_P	SM
35	SM 36:0	[M + H]^+^	10.38	C_41_H_85_N_2_O_6_P	SM

### 3.6. XSBO inhibited the inflammatory reaction in AD rats

In addition, elevated SM levels, not only disrupt lipid homeostasis, but also affect the gene expression of sterol response element binding proteins. Specifically, SM inhibits Ras, leading to the downregulation of MAPK/ERK kinase and extracellular signal-regulated kinase *via* the kinase suppressor of Ras [28]. The inflammatory effects of AD are related to the dysregulation of proinflammatory cytokines, such as IL-6 and TNF-α, as well as chemokines and reactive oxygen/nitrogen species. These inflammatory agents exert their effects through three primary signalling pathways: NF-κB, MAPK and JAK-STAT pathways. Utilizing network pharmacological analysis, we assessed the levels of inflammatory cytokines in the rat brain *via* ELISA. The findings revealed an upregulation of IL-1β, TNF-α and NF-κB in the model group compared to the control group (Figure 5e). In contrast, the XSBO group exhibited a downregulation of these markers.

Inflammatory signals are initiated by pathogen-associated molecular patterns or danger-associated molecular patterns that are recognized by specific receptors [29], which subsequently activate inflammatory cells and induce the production of inflammatory mediators. During inflammation, the production of these mediators facilitates the migration of leukocytes into the locally inflamed tissue, thereby amplifying the inflammatory response. These biologically active mediators of inflammation serve as indicators of the extent of neuroinflammation. In this study, alterations in the inflammatory mediators within the prefrontal cortex were tested with targeted metabolomics, serving as indicators of inflammation activation. Calibration curves for 84 inflammatory mediators were constructed at varying concentration levels employing external standard methods (Table S2). Ten chromatographic peaks were randomly selected to determine the relative standard deviation (RSD%) values for retention time and peak area. The validation results, as detailed in Table S3, demonstrated the proposed quantification method’s excellent linearity, precision, repeatability and stability. A total of 50 inflammatory mediators were identified in the prefrontal cortex of rats (Figure 5f). Compared with the control group, 23 differential inflammatory mediators exhibited significant alterations in the model group, with a reversal trend observed following XSBO administration (Table 4). All together, these findings indicate that XSBO administration effectively suppresses the production of various pro-inflammatory mediators through inflammation-related pathway cascades, thereby inhibiting the progression of AD.

**Table 4.** Details of the differential inflammatory mediators.

No.	Compound	Retention time (min)	Quantitative ion pair	Control vs Model	Model vs HXSB
1	6-keto Prostaglandin F1α	4.08	369.4000/207.3900	↓	↑
2	11β-Prostaglandin F2α	5.85	353.2128/308.9700	↓	↑
3	Prostaglandin F2α	6.44	353.4000/193.0000	↓	↑
4	15(R)-Lipoxin A4	8.11	351.5000/217.2460	↓	↑
5	Leukotriene B4	10.73	335.2000/195.1000	↓	↑
6	(±)5,6-DiHTE	13.45	337.5000/145.1100	↓	↑
7	13(S)-HODE	14.12	295.5000/195.3000	↓	↑
8	9(S)-HODE	14.25	295.5000/171.3000	↓	↑
9	(±)9(10)-EpOME	14.26	295.5000/171.3000	↓	↑
10	(±)9-HODE	14.27	295.4681/171.2429	↓	↑
11	(±)15-HETE	14.53	319.4043/219.2000	↓	↑
12	15(S)-HETE	14.54	319.5000/175.3400	↓	↑
13	(±)11-HETE	14.71	293.5000/113.3000	↓	↑
14	11(S)-HETE	14.91	319.4043/167.2759	↓	↑
15	12(S)-HETE	15.18	319.5000/179.2500	↓	↑
16	(±)12-HETE	15.18	319.4043/179.3011	↓	↑
17	(±)8-HETE	15.22	319.4043/155.2506	↓	↑
18	8(S)-HETE	15.23	319.4681/163.2100	↓	↑
19	5(S)-HETE	15.68	319.4700/203.4000	↓	↑
20	(±)14(15)-EET	16.22	319.5000/219.0000	↓	↑
21	(±)10(11)-EpDPA	16.65	343.5000/153.2600	↓	↑
22	(±)8(9)-EET	16.96	319.5000/154.9500	↓	↑
23	Eicosapentaenoic acid	18.57	301.5000/301.5000	↓	↑

### 3.7. XSBO inhibits Aβ25-35-induced MAPK pathway in SH-SY5Y cells

**Figure 6.**
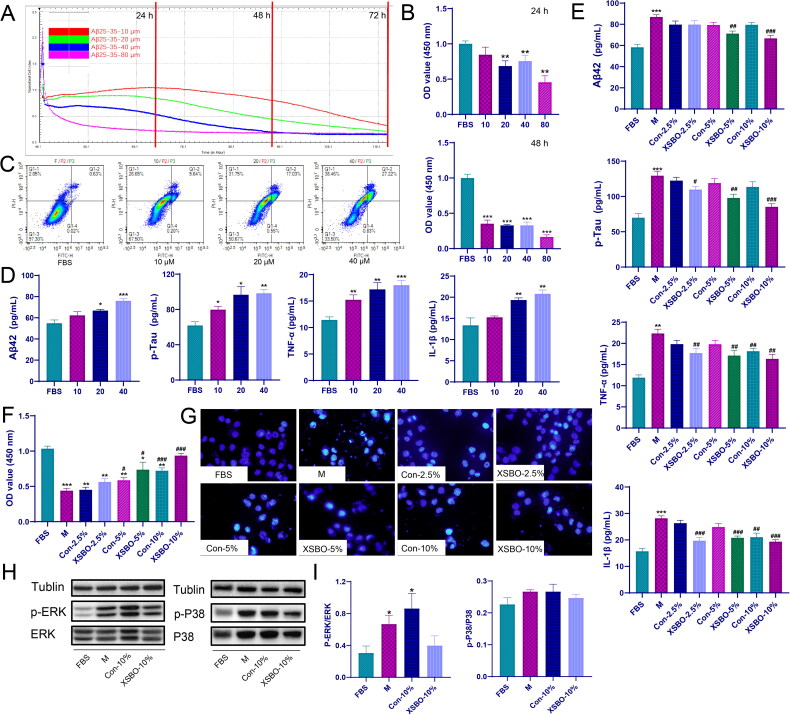
(a) Cell index measured using impedance-based xCELLigence real-time cell analyser; (b) The effect of different concentrations of Aβ25-35 on SH-SY5Y cell viability was determined by CCK-8 assay; (c) The results of apoptosis by flow cytometry; (d) Levels of Aβ42, p-tau, TNF-α, IL-1β under different concentrations of Aβ25-35; (e) Levels of Aβ42, p-tau, TNF-α, IL-1β in treated cell lysates quantified by ELISA; (f) CCK8 assay of different concentrations XSBO on SH-SY5Y cells; (g) Hoechst33258 staining results; (h) The effects of XSBO on the MAPK signaling pathway in SH-SY5Y cells. (i) Quantification of western blot. *p < 0.05, **p < 0.01, ***p < 0.001 versus the FBS group; #*p* < 0.05, ##*p* < 0.01, ###*p* < 0.001 versus the M group.

To further substantiate the role of MAPK pathways in AD, an *in vitro* AD cell model was developed using SH-SY5Y cells induced by Aβ25-35. Exposure to Aβ25-35 at concentrations of 10, 20, 40 and 80 μM led to a dose- and time-dependent reduction in SH-SY5Y cell viability. Based on cell index values (Figure 6a), CCK-8 assays (Figure 6b), flow cytometry analysis (Figure 6c) and model evaluation indicators (Figure 6d), 20 μM of Aβ25-35 was chosen as optimum dose for subsequent experiments. In comparison to the control group, Aβ25-35 stimulation significantly elevated the levels of Aβ42, phosphorylated Tau (p-Tau) and the pro-inflammatory cytokines TNF-α and IL-1β (Figure 6e), thereby intensifying cellular injury and apoptosis (Figure 6f-g). Conversely, treatment with XSBO serum effectively reduced the levels of Aβ42 and p-Tau, attenuated inflammation, and restored the proliferative capacity of SH-SY5Y cells, with the 10% drug-containing serum group exhibiting the most significant effect. Western blot analysis further corroborated that XSBO alleviates Aβ25-35 induced neuroinflammation. XSBO treatment resulted in decreased expression of phosphorylated P38 (p-P38) and phosphorylated ERK (p-ERK) (Figure 6h-i), suggesting that the therapeutic efficacy of XSBO in AD may be attributed to its ability to mitigate inflammation through inhibition of the MAPK pathway.

## 4. Discussion

Although XSBO has demonstrated potential anti-AD biological activities, the precise mechanisms by which XSBO exerts its effects against AD remain largely unexplored. Thus, this study utilized a combination of network pharmacology and molecular docking to systematically investigate the active constituents, core targets and key signalling pathways involved in the anti-AD effects of XSBO. Concurrently, animal experiments were performed to validate the reliability of the network pharmacology findings.

**Figure 7.**
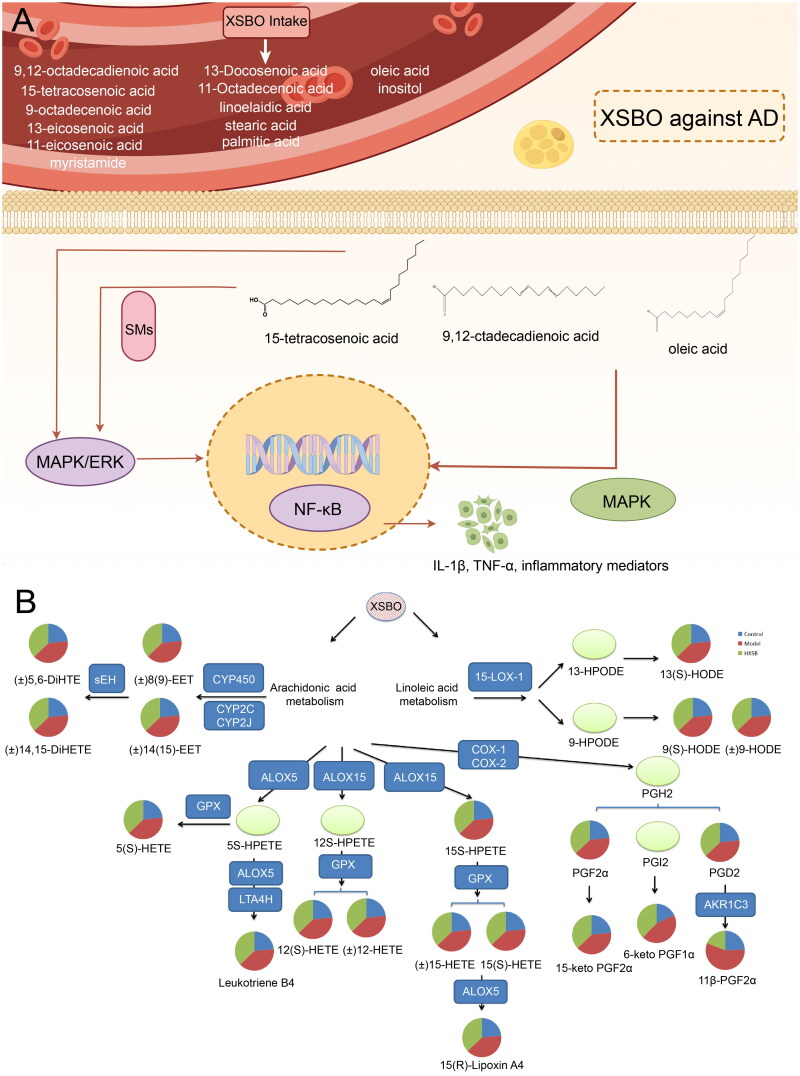
(a) Mechanism diagram of XSBO against AD (drawn by Figdraw 2.0, ID:STYPT9f28d); (b) Metabolic pathways for inflammatory mediators.

Network pharmacology analysis revealed that the active constituents in XSBO, such as 9,12-octadecadienoic acid, linoelaidic acid, 11-octadecenoic acid, oleic acid and 15-tetracosenoic acid, interact with targets including MAPK1, MAPK3, AKT1, RXRA, RXRB, PPARD and PPARA to modulate inflammation-related signalling pathways and the sphingolipid signalling pathway. Previous studies have indicated that the MAPK pathway may be activated by fatty acids under various physiological conditions. The MAPK signalling pathway is implicated in the regulation of inflammation and fatty acid metabolism. For instance, oleic acid has been demonstrated to activate P38-MAPK in rat hepatoma dRLh-84 cells [30]. The MAPK pathway is implicated in synaptic plasticity and the pathogenesis of neurodegenerative disorders, underscoring its potential as a therapeutic target for AD [31]. Several unsaturated fatty acids, including 9,12-octadecadienoic acid and oleic acid, have also been shown to activate PPARs [31–33]. PPARs are crucial in regulating gene expression and are involved in modulating various signalling pathways, such as MAPK, NF-κB and JAK/STAT, to regulate inflammatory and immune responses associated with neurological disorders [34,35]. Notably, 9,12-octadecadienoic acid, the primary precursor to arachidonic acid, exhibits significant inhibitory effects on microglial cell activation, potentially reversing the inflammatory response induced by palmitic acid treatment [36,37]. Consequently, PPARα emerges as a potential sensor for free fatty acids in the brain. Retinoid X receptors (RXRs), including RXRA, RXRB and RXRG, function in conjunction with retinoic acid receptors (RARs), namely RARA, RARB and RARG, as nuclear retinoid receptors. RXRA plays a critical role in cellular senescence and lipid homeostasis. The knockdown of RXRA induces senescence through the activation of the ITPR2/MCU/ROS/DNA damage/p53/p21 signalling cascade, whereas its overexpression yields contrary effects [38]. Considering the complex pathological mechanisms implicated in AD, employing multitargeted therapeutic agents offers a promising approach for the development of more effective treatment strategies (Figure 7a).

KEGG enrichment analysis has identified several crucial pathways, such as the inflammatory mediator regulation of TRP channels, the arachidonic acid metabolism, the PPAR signalling pathway, the AMPK signalling pathway, and the JAK-STAT signalling pathway, all of which are closely linked to the pathogenesis of AD. TRP channels are widely expressed across various brain cell types and play a pivotal role in the development of numerous neuroinflammatory disorders [39]. Previous research has shown that the inhibition of TRP channels, including TRPV2 and TRPM4, can mitigate cognitive deficits in a range of disease contexts models [40,41]. The activation of AMPK has been demonstrated to enhance neuronal survival following exposure to Aβ peptides, inhibit the accumulation of phosphorylated Tau, and promote autophagy-dependent degradation of Aβ [42,43]. Shah et al. reported that the AMPK/SIRT1 signalling pathway plays a role in regulating cognitive functions and Aβ deposition in AD rat models [44]. The JAK-STAT pathway is widely recognized for its role in immune cell function, particularly in response to growth factors and cytokines [45]. Additionally, it is well-established that the arachidonic acid metabolism pathway acts as a mechanism for initiating inflammatory responses in various immune cells [46]. Certain polyunsaturated fatty acids, including oleic, palmitic and linoleic acids [47,48] along with arachidonic acid metabolites [49], are considered natural ligands of PPAR. Generally, activated PPARs exert negative regulation on inflammatory gene expression through the following mechanisms: (1) direct interaction with NF-κB; and (2) inhibition of JNK by PPAR-γ and RXR-α ligands, which subsequently reduces the phosphorylation of c-Jun and P38 MAPK. Research has demonstrated that toll-like receptors, particularly TLR4, are transmembrane proteins that play a significant role in initiating the inflammatory response by recognizing specific ligands [50]. The TLR4-mediated signalling pathway triggers the activation of downstream signalling cascades, including NF-κB and MAPK, which regulate the secretion of proinflammatory mediators and cytokines [51]. The activation of MAPK-ERK1/2 is associated with neurofibrillary tangles in AD [52], and modulation of P38α MAPK may potentially reduce beta-amyloid production, tau hyperphosphorylation, synaptic dysfunction and behavioural deficits [53–55]. *In vitro* experiments further confirmed that XSBO exerts its anti-inflammatory effect through the MAPK pathway.

During the inflammatory response, unsaturated fatty acids in XSBO are linked to lipid membranes, from which they are released by phospholipase A2 and subsequently processed into various biologically active signalling molecules. These include prostaglandins, thromboxanes, lipoxins and leukotrienes. These molecules possess both pro- and anti-apoptotic properties, play significant roles in inflammation, modulate immune responses, and are involved in other yet-to-be-identified processes. As such, this study systematically characterized the profiles of inflammatory mediators in the AD rat brains, and elucidated the regulatory effects of XSBO on inflammatory functions. A total of 23 differential inflammatory mediators were identified as characteristic markers in AD rats treated with XSBO. Notably, these mediators were predominantly associated with the metabolic processes of arachidonic and linoleic acids (Figure 7b). The oxylipins 9-HODE and 13-HODE, derived from linoleate through the lipoxygenase pathway [56], are recognized as potential biomarkers for oxidative stress and inflammation [57]. Arachidonic acid, a pivotal inflammatory intermediate, gives rise to various inflammatory mediators, including prostaglandins, thromboxanes and leukotrienes, *via* the lipoxygenase, cyclooxygenase and cytochrome P450 pathways [58]. Prostaglandins, a critical class of pro-inflammatory mediators, such as prostaglandin D2, prostaglandin F2α, and prostaglandin I2, along with their major metabolites, are abundantly present in the brain. These compounds play significant roles in inflammation, neuronal restoration and the progression of AD [59–62]. Hydroxyeicosatetraenoic acids (HETEs) are notable as precursors to metabolites such as leukotrienes, lipoxins, trioxilins and eoxins, and also function as signalling molecules independently [63]. ALOX15 is one of the most prevalent lipoxygenase isoforms in the central nervous system, present in both neurons and glial cells, with 12-HETE and 15-HETE being the primary products [64]. ALOX5 facilitates the conversion of arachidonic acid to 5-HPETE, which is subsequently rapidly transformed into other products. The release of 5-HPETE to ubiquitous cellular glutathione peroxidases results in its reduction to 5-HETE [65]. Treatment of the N2A-APPswe cell line, a murine cell line expressing human APP with the AD-prone Swedish mutation, with 5-HETE significantly increased Aβ production [66]. Moreover, ALOX5 may convert the transient 5-HPETE to leukotriene A4, the precursor for the synthesis of other leukotrienes. The highly unstable leukotriene A4 can undergo hydrolysis to form leukotriene B4. The pro-inflammatory leukotriene B4 is among the most potent chemotactic molecules known, inducing the recruitment and activation of monocytes, neutrophils and eosinophils [67]. The treatment of cultured monocytes with LTB4 has been demonstrated to activate the MAPK and PI3/AKT signalling pathways, leading to the overproduction of IL-6, monocyte chemoattractant protein 1 and TNF-α. Epoxyeicosatrienoic acids (EETs), synthesized from arachidonic acid *via* the cytochrome P450 pathway, are active endogenous molecules associated with inflammation and oxidative stress [68]. These inflammatory mediators play a crucial role in the inflammatory response and are integral to the pathology of AD. A comprehensive understanding of the signalling pathways of these inflammation mediators is essential for elucidating the underlying mechanisms and developing potential therapeutic strategies for AD. Collectively, these findings indicate that the administration of XSBO suppresses the production of various pro-inflammatory mediators through the modulation of inflammation-related signalling cascades, thereby inhibiting the progression of AD.

Moreover, XSBO contains a distinctive fatty acid, 15-tetracosenoic acid, which is known to be linked to the sphingosine group *via* an amide bond, thereby facilitating the formation of glycosphingolipid and sphingomyelin. Sphingolipids, including ceramide and sphingomyelin, are a class of lipids characterized by an extended sphingoid base backbone [69]. It has been suggested that ceramide plays a crucial role in AD pathophysiology by influencing Aβ generation and tau protein hyperphosphorylation. Vozella et al. reported an age-associated increase in nervonic acid-containing sphingolipids and the enzyme responsible for nervonic acid synthesis in the ageing hippocampus of both male and female mice, indicating that the accumulation of these lipids may contribute to brain ageing [70]. Inhibition of sphingomyelinase, the enzyme responsible for catalyzing the hydrolysis of sphingomyelin to ceramide, leads to γ-secretase activity and consequently decreased Aβ production [71]. Elevated ceramide levels activate initiate apoptotic pathways, resulting in neuronal loss and ultimately worsening symptoms in individuals with AD. In the early stages of AD, an increase in ceramide levels is observed within the frontal and temporal cortices, accompanied by a concomitant decrease in sphingomyelin levels. These studies indicate that dysregulated sphingolipid metabolism plays a significant role in the initial phases of AD, as elevated levels of ceramide levels contribute to lipid peroxidation, oxidative stress, mitochondrial impairment and neuronal apoptosis. Moreover, increased sphingomyelins levels have been shown to affect lipid homeostasis and the gene expression of sterol response element binding proteins. SMs inhibit Ras, leading to the suppression of MAPK/ERK kinase and extracellular signal-regulated kinase *via* kinase suppressor of Ras [28]. The findings suggest that the sphingolipid metabolic equilibrium in rat models of AD model is disrupted. Similarly, the decrease in SMs levels supports the proposed role of XSBO in the degradation of sphingolipids.

## 5. Conclusion

In conclusion, this study employed an integrative approach combining network pharmacology, molecular docking and experimental validation to elucidate the pharmacological basis and potential mechanisms of XSBO in the context of AD. The active constituents of XSBO, such as 9,12-octadecadienoic acid, linoelaidic acid, 11-octadecenoic acid and 13-octadecenoic acid, were found to interact with targets including MAPK1, MAPK3, AKT1, RXRA, RXRB, PPARD and PPARA. These interactions facilitate the comprehensive modulation of inflammation-related signalling pathways and the sphingolipid signalling pathway. *In vitro* experiments demonstrated that XSBO mitigates cell injury and inflammation associated with AD in SH-SY5Y cells by inhibiting MAPK signalling pathways. Overall, this study provides a scientific framework for understanding the potential mechanisms of XSBO in AD treatment, thereby laying a theoretical foundation for its clinical application.

## Supplementary Material

Supplymentary materials.docx

## Data Availability

Raw metabolomics data have been deposited in NGDC OMIX database and are accessible through OMIX ID OMIX009342 (https://ngdc.cncb.ac.cn/omix/preview/TnIqFD62). Further details can be requested from the corresponding author.
