# Supplementary material for: Study on the mechanism of Xanthoceras sorbifolia Bunge oil in the treatment of Alzheimer’s disease by an integrated “network pharmacology-metabolomics” strategy
Source: Ann Med. 2025 May 8;57(1):2499700. doi: 10.1080/07853890.2025.2499700 (PMC12064105; doi:10.1080/07853890.2025.2499700)
Supplement: Supplymentary materials.docx [file IANN_A_2499700_SM0964.docx]

Table S1 Compound information of inflammatory mediator mixed standards

| NO. | Product name | Product number | Standards | CAS | Retention time（min） |
| --- | --- | --- | --- | --- | --- |
| 1 | Arachidonic Acid CYP450 Metabolite LC-MS Mixture | 20665 | (±)14,15-DiHETE | 77667-09-5 | 11.83 |
|  |  |  | 20-HETE | 79551-86-3 | 13.33 |
|  |  |  | (±)14(15)-EET | 74868-37-4 | 16.22 |
|  |  |  | (±)11(12)-EET | 123931-40-8 | 16.75 |
|  |  |  | (±)8(9)-EET | 81246-85-7 | 16.96 |
|  |  |  | (±)11(12)-DiHET | 192461-95-3 | 12.38 |
|  |  |  | (±)8(9)-DiHET | 192461-96-4 | 12.84 |
|  |  |  | (±)5(6)-DiHET | 213382-49-1 | 13.45 |
| 2 | Primary Prostaglandin Metabolite LC-MS Mixture | 19300 | 15-keto Prostaglandin E2 | 26441-05-4 | 7.37 |
|  |  |  | 13,14-dihydro-15-keto Prostaglandin E2 | 363-23-5 | 8.05 |
|  |  |  | 15-keto Prostaglandin F2α | 27376-76-7 | 7.05 |
|  |  |  | 11β-Prostaglandin F2α | 38432-87-0 | 5.85 |
|  |  |  | 13,14-dihydro-15-keto Prostaglandin D2 | 59894-07-4 | 8.79 |
|  |  |  | Prostaglandin J2 | 60203-57-8 | 9.41 |
|  |  |  | 15-deoxy-delta 12,14-Prostaglandin J2 | 87893-55-8 | 13.28 |
| 3 | Leukotriene B4 Pathway LC-MS Mixture | 22640 | 18-carboxy dinor Leukotriene B4 | 102674-12-4 | 13.43 |
|  |  |  | 20-carboxy Leukotriene B4 | 80434-82-8 | 4.33 |
|  |  |  | Leukotriene B4 | 71160-24-2 | 10.73 |
|  |  |  | 5(S)-HETE | 70608-72-9 | 15.68 |
| 4 | Oxidized Lipid HPLC Mixture | 34004 | (±)9-HODE | 98524-19-7 | 14.27 |
| 5 | Cysteinyl Leukotriene HPLC Mixture II | 20002 | Leukotriene C4 | 72025-60-6 | 9.16 |
|  |  |  | Leukotriene D4 | 73836-78-9 | 8.87 |
|  |  |  | Leukotriene E4 | 75715-89-8 | 9.34 |
|  |  |  | N-acetyl Leukotriene E4 | 80115-95-3 | 12.08 |
|  |  |  | Leukotriene F4 | 83851-42-7 | 9.45 |
|  |  |  | Prostaglandin B2 | 13367-85-6 | 9.4 |
| 6 | Polyunsaturated Fatty Acid LC-MS Mixture | 17941 | α-Linolenic Acid | 463-40-1 | 19.04 |
|  |  |  | Dihomo γ Linolenic Acid | 1783-84-2 | 21.26 |
|  |  |  | Eicosapentaenoic Acid | 10417-94-4 | 18.57 |
|  |  |  | Stearidonic Acid | 20290-75-9 | 17.39 |
|  |  |  | Docosapentaenoic Acid | 24880-45-3 | 20.51 |
|  |  |  | Adrenic Acid | 28874-58-0 | 22 |
|  |  |  | (±)12,13-DiHOME | 263399-35-5 | 11.03 |
| 7 | Linoleic Acid Oxylipins LC-MS Mixture | 20794 | (±)9,10-DiHOME | 263399-34-4 | 11.4 |
|  |  |  | 9(S)-HODE | 73543-67-6 | 14.25 |
|  |  |  | 13(S)-HODE | 29623-28-7 | 14.12 |
|  |  |  | 13-OxoODE | 54739-30-9 | 14.71 |
|  |  |  | 9-OxoODE | 54232-59-6 | 15.11 |
|  |  |  | (±)12(13)-EpOME | 503-07-1 | 14.13 |
|  |  |  | (±)9(10)-EpOME | 61949-82-4 | 14.26 |
|  |  |  | Prostaglandin E2 | 363-24-6 | 6.8 |
| 8 | Primary COX and LOX LC-MS Mixture | 19101 | Prostaglandin F2α | 551-11-1 | 6.44 |
|  |  |  | Prostaglandin D2 | 41598-07-6 | 6.79 |
|  |  |  | 12(S)-HETE | 54397-83-0 | 15.18 |
|  |  |  | 12(S)-HHTrE | 54397-84-1 | 12.01 |
|  |  |  | Thromboxane B2 | 54397-85-2 | 5.74 |
|  |  |  | 15(S)-HETE | 54845-95-3 | 14.54 |
|  |  |  | 6-keto Prostaglandin F1α | 58962-34-8 | 4.08 |
|  |  |  | 9(S)-HOTrE | 89886-42-0 | 12.77 |
| 9 | ALA and GLA Oxylipin LC-MS Mixture | 22638 | 13(S)-HOTrE(γ) | 4784-20-6 | 13.17 |
|  |  |  | (±)5-HETE | 73307-52-5 | 15.68 |
| 10 | (±)-HETE HPLC Mixture | 34002 | (±)8-HETE | 79495-84-4 | 15.22 |
|  |  |  | (±)11-HETE | 73804-65-6 | 14.91 |
|  |  |  | (±)12-HETE | 71030-37-0 | 15.18 |
|  |  |  | (±)15-HETE | 73836-87-0 | 14.53 |
|  |  |  | Resolvin E1 | 552830-51-0 | 4.31 |
| 11 | SPM E-series LC-MS Mixture | 19417 | （±)18-HEPE | 141110-17-0 | 12.94 |
|  |  |  | 11(S)-HETE | 54886-50-9 | 14.91 |
| 12 | Arachidonic Acid Oxylipin LC-MS Mixture | 20666 | 15-OxoETE | 81416-72-0 | 15.05 |
|  |  |  | 8(S)-HETE | 98462-03-4 | 15.23 |
|  |  |  | 9(R)-HETE | 107656-14-4 | 15.19 |
|  |  |  | 12-OxoETE | 108437-64-5 | 15.05 |
|  |  |  | 5-OxoETE | 106154-18-1 | 16.68 |
|  |  |  | Resolvin D2 | 82864-77-5 | 7.35 |
| 13 | SPM D-series LC-MS Mixture | 18702 | Resolvin D5 | 578008-43-2 | 10.66 |
|  |  |  | Resolvin D1 | 872993-05-0 | 8.1 |
|  |  |  | Resolvin D3 | 916888-47-6 | 7.13 |
|  |  |  | 17(R)-Resolvin D1 | 528583-91-7 | 8.11 |
|  |  |  | Lipoxin A4 | 89663-86-5 | 8.11 |
| 14 | Lipoxin LC-MS Mixture | 19412 | Lipoxin B4 | 98049-69-5 | 7.14 |
|  |  |  | 15(R)-Lipoxin A4 | 506-32-1 | 8.11 |
|  |  |  | (±)19,20-DiHDPA | 1350373-35-1 | 11.82 |
| 15 | Docosahexaenoic Acid CYP450 Oxylipins LC-MS Mixture | 22639 | (±)16(17)-DiHDPA | 1345275-27-5 | 12.24 |
|  |  |  | (±)13,14-DiHDPA | 1345275-24-2 | 12.43 |
|  |  |  | (±)10,11-DiHDPA | 1345275-22-0 | 12.71 |
|  |  |  | (±)7,8-DiHDPA | 168111-93-1 | 13.27 |
|  |  |  | (±)16(17)-EpDPA | 155073-46-4 | 16.42 |
|  |  |  | (±)13(14)-EpDPA | 895127-64-7 | 16.53 |
|  |  |  | (±)10(11)-EpDPA | 895127-65-8 | 16.65 |
|  |  |  | 5(S)-HEPE | 92008-51-0 | 14.15 |
| 16 | ω-3 Hydroxy Acid HPLC Mixture | 34003 | 12(S)-HEPE | 116180-17-7 | 13.75 |
|  |  |  | 15(S)-HEPE | 86282-92-0 | 13.41 |
|  |  |  | 13(S)-HOTrE | 87984-82-5 | 12.97 |
|  |  |  | 15(S)-HEtrE | 92693-02-2 | 15.34 |

Table S2 Standard curves of inflammatory mediators

| No. | Compound | Retention time (min) | Standard curve | Linearity Range (ng/mL) | R2 |
| --- | --- | --- | --- | --- | --- |
| 1 | 6-keto Prostaglandin F1α | 4.08 | y = 154.51x + 55.307 | 0.78-50 | 0.9962 |
| 2 | Resolvin E1 | 4.31 | y = 23.49x - 131.47 | 5-100 | 0.9915 |
| 3 | 20-carboxy Leukotriene B4 | 4.33 | y = 11.085x - 268.54 | 25-400 | 0.9998 |
| 4 | Thromboxane B2 | 5.74 | y = 112.87x - 59.56 | 1.56-200 | 0.9973 |
| 5 | 11β-Prostaglandin F2α | 5.85 | y = 112.28x - 20.391 | 6.25-400 | 0.9961 |
| 6 | Prostaglandin F2α | 6.44 | y = 260.06x + 983.09 | 1.56-200 | 0.9982 |
| 7 | Prostaglandin D2 | 6.79 | y = 348.83x - 69.095 | 0.2-100 | 0.9982 |
| 8 | Prostaglandin E2 | 6.80 | y = 1.9253x + 14.375 | 12.5-400 | 0.9921 |
| 9 | 15-keto Prostaglandin F2α | 7.05 | y = 304.71x + 151.1 | 1.56-100 | 0.9987 |
| 10 | Resolvin D3 | 7.13 | y = 181.71x + 0.6711 | 0.31-75 | 0.9979 |
| 11 | Lipoxin B4 | 7.14 | y = 96.378x - 2.087 | 0.31-10 | 0.9939 |
| 12 | Resolvin D2 | 7.35 | y = 2.4644x - 1.6949 | 0.63-20 | 0.9984 |
| 13 | 15-keto Prostaglandin E2 | 7.37 | y = 14.133x + 14.362 | 25-400 | 0.9957 |
| 14 | 13,14-dihydro-15-keto Prostaglandin E2 | 8.05 | y = 140.97x + 310.94 | 3.13-400 | 0.9989 |
| 15 | Resolvin D1 | 8.10 | y = 16.733x - 3.5392 | 0.63-75 | 0.9971 |
| 16 | 17(R)-Resolvin D1 | 8.11 | y = 44.813x - 33.54 | 1.25-75 | 0.9976 |
| 17 | 15(R)-Lipoxin A4 | 8.11 | y = 211.26x + 16.305 | 0.31-50 | 0.9959 |
| 18 | Lipoxin A4 | 8.11 | y = 17.273x + 1.9996 | 1.25-25 | 0.9998 |
| 19 | 13,14-dihydro-15-keto Prostaglandin D2 | 8.79 | y = 104.16x - 36.612 | 3.125-200 | 0.9977 |
| 20 | Leukotriene D4 | 8.87 | y = 111.56x - 176.23 | 2.61-83.34 | 0.9981 |
| 21 | Leukotriene C4 | 9.16 | y = 115.17x + 503.13 | 5.21-333.35 | 0.9987 |
| 22 | Leukotriene E4 | 9.34 | y = 109.44x + 1114.4 | 5.21-666.67 | 0.9985 |
| 23 | Prostaglandin B2 | 9.40 | y = 487.24x - 82.141 | 0.31-41.67 | 0.9974 |
| 24 | Prostaglandin J2 | 9.41 | y = 42.161x + 147.39 | 6.25-400 | 0.9989 |
| 25 | Leukotriene F4 | 9.45 | y = 52.012x + 111.11 | 5.21-333.35 | 0.9986 |
| 26 | Resolvin D5 | 10.66 | y = 31.922x - 17.409 | 0.31-20 | 0.9944 |
| 27 | Leukotriene B4 | 10.73 | y = 221.29x - 30.776 | 0.78-50 | 0.9975 |
| 28 | (±)12,13-DiHOME | 11.03 | y = 345.15x - 3.6527 | 0.2-50 | 0.9984 |
| 29 | (±)9,10-DiHOME | 11.40 | y = 25.711x + 97.216 | 3.13-400 | 0.9954 |
| 30 | (±)19,20-DiHDPA | 11.82 | y = 67.121x + 8.1212 | 1.56-50 | 0.9929 |
| 31 | (±)14,15-DiHETE | 11.83 | y = 202.41x + 62.357 | 0.39-25 | 0.9971 |
| 32 | 12(S)-HHTrE | 12.01 | y = 21.196x - 80.91 | 3.13-400 | 0.9969 |
| 33 | N-acetyl Leukotriene E4 | 12.08 | y = 190x - 1.2769 | 1.3-83.34 | 0.999 |
| 34 | (±)16,17-DiHDPA | 12.24 | y = 89.461x - 149.18 | 1.56-25 | 0.9984 |
| 35 | (±)11,12-DiHET | 12.38 | y = 176.13x - 345.8 | 0.78-50 | 0.9968 |
| 36 | (±)13,14-DiHDPA | 12.43 | y = 1133.5x - 151.24 | 0.39-12.5 | 0.9952 |
| 37 | (±)10,11-DiHDPA | 12.71 | y = 462.82x + 12.891 | 1.56-25 | 0.9968 |
| 38 | 9(S)-HOTrE | 12.77 | y = 83.661x + 240.06 | 3.13-400 | 0.9992 |
| 39 | (±)8,9-DiHET | 12.84 | y = 13.827x + 108.59 | 25-800 | 0.9983 |
| 40 | （±)18-HEPE | 12.94 | y = 179.42x - 91.912 | 0.16-100 | 0.9985 |
| 41 | 13(S)-HOTrE | 12.97 | y = 684.51x - 16.465 | 0.39-12.5 | 0.9942 |
| 42 | 13(S)-HOTrE(γ) | 13.17 | y = 411.4x + 375.09 | 3.13-100 | 0.9974 |
| 43 | (±)7,8-DiHDPA | 13.27 | y = 71.709x - 144.9 | 3.13-50 | 0.9946 |
| 44 | 15-deoxy-delta 12,14-Prostaglandin J2 | 13.28 | y = 273.39x + 225.4 | 0.39-50 | 0.9989 |
| 45 | 20-HETE | 13.33 | y = 11.984x - 209.9 | 25-800 | 0.9926 |
| 46 | 15(S)-HEPE | 13.41 | y = 711.81x + 477.56 | 0.39-12.5 | 0.9952 |
| 47 | 18-carboxy dinor Leukotriene B4 | 13.43 | y = 39.385x + 354.42 | 6.25-400 | 0.9973 |
| 48 | (±)5,6-DiHET | 13.45 | y = 341.56x + 32.666 | 0.39-25 | 0.9957 |
| 49 | 12(S)-HEPE | 13.75 | y = 798.9x + 3055.9 | 6.25-100 | 0.997 |
| 50 | 13(S)-HODE | 14.12 | y = 133.24x - 0.377 | 3.13-50 | 0.9983 |
| 51 | (±)12(13)-EpOME | 14.13 | y = 135.71x + 17.936 | 0.39-50 | 0.9945 |
| 52 | 5(S)-HEPE | 14.15 | y = 149.8x + 583.89 | 6.25-100 | 0.995 |
| 53 | 9(S)-HODE | 14.25 | y = 64.32x + 37.368 | 3.13-100 | 0.9964 |
| 54 | (±)9(10)-EpOME | 14.26 | y = 56.146x + 7.314 | 3.13-200 | 0.9991 |
| 55 | (±)9-HODE | 14.27 | y = 214.66x + 189.19 | 1.56-200 | 0.9987 |
| 56 | (±)15-HETE | 14.53 | y = 1248x - 132.74 | 0.39-25 | 0.9961 |
| 57 | 15(S)-HETE | 14.54 | y = 268.86x + 13.327 | 0.1-25 | 0.9964 |
| 58 | 13-OxoODE | 14.71 | y = 21.075x - 64.712 | 6.25-200 | 0.9935 |
| 59 | (±)11-HETE | 14.91 | y = 983.28x - 305.76 | 3.13-50 | 0.9985 |
| 60 | 11(S)-HETE | 14.91 | y = 1098.2x + 835.46 | 0.78-50 | 0.9996 |
| 61 | 12-OxoETE | 15.05 | y = 9.2523x + 48.824 | 12.5-200 | 0.9953 |
| 62 | 15-OxoETE | 15.05 | y = 36.299x + 11.381 | 3.125-200 | 0.9977 |
| 63 | 9-OxoODE | 15.11 | y = 150.81x + 18.742 | 0.39-25 | 0.999 |
| 64 | 12(S)-HETE | 15.18 | y = 1358.9x - 102.08 | 0.2-12.5 | 0.9994 |
| 65 | (±)12-HETE | 15.18 | y = 298.47x - 190.28 | 0.78-50 | 0.9989 |
| 66 | 9(R)-HETE | 15.19 | y = 29.302x + 19.711 | 3.125-100 | 0.9902 |
| 67 | (±)8-HETE | 15.22 | y = 285.57x + 36.326 | 0.39-25 | 0.9959 |
| 68 | 8(S)-HETE | 15.23 | = 212.38x + 130.78 | 0.39-50 | 0.9955 |
| 69 | 15(S)-HEtrE | 15.34 | y = 1988.4x + 1594.7 | 0.39-50 | 0.9991 |
| 70 | 5(S)-HETE | 15.68 | y = 8.3457x - 294.11 | 50-400 | 0.9915 |
| 71 | (±)5-HETE | 15.68 | y = 1125.6x - 135.81 | 0.39-25 | 0.998 |
| 72 | (±)14(15)-EET | 16.22 | y = 98.785x + 405.88 | 6.25-400 | 0.999 |
| 73 | (±)16(17)-EpDPA | 16.42 | y = 7.6219x - 18.17 | 1.56-100 | 0.9858 |
| 74 | (±)13(14)-EpDPA | 16.53 | y = 20.144x + 113.37 | 3.13-200 | 0.9842 |
| 75 | (±)10(11)-EpDPA | 16.65 | y = 89.586x + 45.36 | 1.56-100 | 0.9977 |
| 76 | 5-OxoETE | 16.68 | y = 190.88x + 23.306 | 0.78-50 | 0.998 |
| 77 | (±)11(12)-EET | 16.75 | y = 209.79x + 36.997 | 0.78-200 | 0.9989 |
| 78 | (±)8(9)-EET | 16.96 | y = 84.792x - 453.57 | 12.5-400 | 0.996 |
| 79 | Stearidonic Acid | 17.39 | y = 8.0183x - 86.627 | 25-800 | 0.9926 |
| 80 | Eicosapentaenoic Acid | 18.57 | y = 40.947x + 736.69 | 3.125-800 | 0.9984 |
| 81 | α-Linolenic Acid | 19.04 | y = 704.58x + 547.91 | 0.2-100 | 0.9933 |
| 82 | Docosapentaenoic Acid | 20.51 | y = 1059.3x + 2376.3 | 0.2-100 | 0.9963 |
| 83 | Dihomo γ Linolenic Acid | 21.26 | y = 641.82x + 1572.8 | 3.13-100 | 0.9979 |
| 84 | Adrenic Acid | 22.00 | y = 112.68x + 153.87 | 3.13-200 | 0.9995 |

Table S3 The results of precision, stability, and repeatability of inflammatory mediators

| No. | Precision RSD% | | Stability RSD% | | Repeatability RSD% | |
| --- | --- | --- | --- | --- | --- | --- |
|  | Retention time | Peak area | Retention time | Peak area | Retention time | Peak area |
| 1 | 0.10 | 5.70 | 0.04 | 10.25 | 0.11 | 21.30 |
| 2 | 0.07 | 3.74 | 0.07 | 11.44 | 0.13 | 19.19 |
| 3 | 0.04 | 7.18 | 0.07 | 7.60 | 0.07 | 17.16 |
| 4 | 0.06 | 9.28 | 0.05 | 13.74 | 0.07 | 20.09 |
| 5 | 0.08 | 7.73 | 0.05 | 7.11 | 0.03 | 18.60 |
| 6 | 0.04 | 5.22 | 0.07 | 11.69 | 0.05 | 21.67 |
| 7 | 0.07 | 5.32 | 0.06 | 14.12 | 0.12 | 16.52 |
| 8 | 0.03 | 6.09 | 0.13 | 6.72 | 0.13 | 22.45 |
| 9 | 0.08 | 6.12 | 0.05 | 7.51 | 0.09 | 23.16 |
| 10 | 0.05 | 3.50 | 0.07 | 11.95 | 0.09 | 19.43 |
